# Technical consideration of the MOVARPE technique in intricate pectus excavatum deformity

**DOI:** 10.1007/s00508-017-1214-y

**Published:** 2017-05-24

**Authors:** Anton H. Schwabegger, Barbara Del Frari, Julia Metzler

**Affiliations:** Department of Plastic, Reconstructive and Aesthetic Surgery, Medical University Innsbruck, 6020 Innsbruck, Austria

**Keywords:** Pectus excavatum, Adult, Pectus bar, MOVARPE, Surgery

## Abstract

**Background:**

For the correction of pectus excavatum (PE) deformities in adolescents, adults, and generally in asymmetric cases, a semi-open approach called the MOVARPE (minor open videoendoscopically assisted repair of pectus excavatum) technique is used, consisting of standard pectus bar implantation hybridized with auxiliary sternum osteotomy and multiple chondrotomies. In this study, we report our experiences, discuss pros and cons, and provide technical refinements.

**Methods:**

Between September 2005 and March 2015, 61 patients were selected to undergo the MOVARPE instead of the standard MIRPE (minimally invasive repair of pectus excavatum) procedure because of age or specific morphologic characteristics of PE. Patient age ranged from 14 to 45 years (mean 23.4 years).

**Results:**

Auxiliary incisions for skeletal relaxation enabled symmetric remodeling and, in most cases, circumvented the need for a second pectus bar. The bars were left in position for a mean of 19.3 months (range: 12 to 35 months). There were no major complications. Minor complications such as pleural effusion, temporary pneumothorax, and mild recurrence of the deformity after bar removal were seen at rates similar to those for standard techniques. In the current study reporting outcomes of the previously described MOVARPE procedure, the authors saw no evidence of a possible disadvantage in the overall concept or execution of the procedure for the suggested indication.

**Conclusion:**

From this experience, we can state that, as an alternative to the MIRPE technique, MOVARPE is a method that offers high efficacy, particularly for rigid and complex pectus excavatum deformities at or beyond puberty.

## Introduction

Currently, the minimally invasive repair of pectus excavatum (MIRPE) technique is the preferred method in children and prepubescent patients [1–3]. A curved stainless steel bar is inserted behind the sternum through the chest cavity with the convex surface facing down and then rotated 180 degrees to elevate the anterior thoracic wall, thus correcting the deformity and avoiding additional surgery. The literature contains a wealth of information on the successful application of this technique in children. However, the correction of complicated cases like steeply sloping or asymmetric deformities, as well as correction in adolescents, adults, or athletic patients with matured and thus rigid skeletal structures remains controversial [4–7].

The goal of this recent 10-year report was to determine whether the high success rate of our MOVARPE technique (minor open videoendoscopically assisted repair of pectus excavatum), a modification of the MIRPE technique, can be confirmed by the long-term outcome score devised by Goretsky et al. [8] and, if so, to describe the technique in detail. As a semi-open access technique, it hybridizes the basic concepts of the open Ravitch procedure and the minimally invasive MIRPE technique [1, 9, 10].

## Patients and methods

**Fig. 1:**
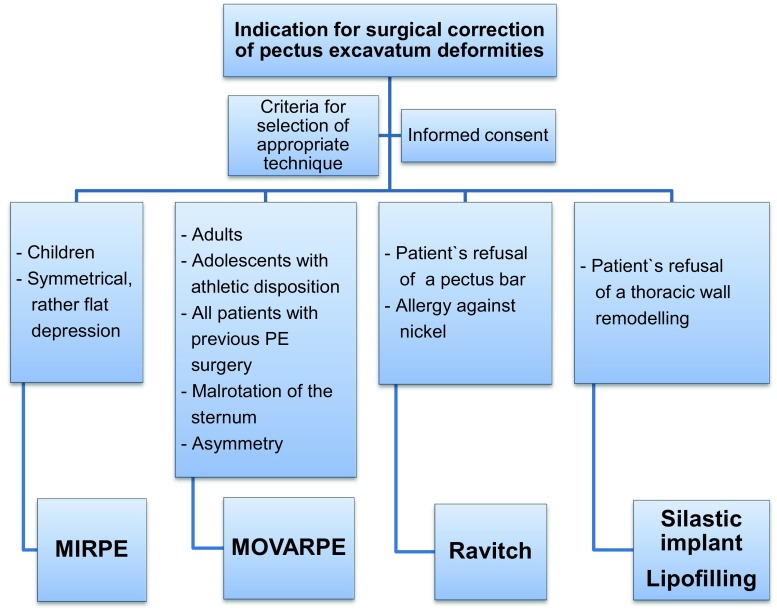
Our algorithm for surgical correction of pectus excavatum deformity. *PE* pectus excavatum, *MIRPE* minimally invasive repair of pectus excavatum, *MOVARPE* minor open videoendoscopically assisted repair of pectus excavatum

In a retrospective study, we analyzed 145 patients who underwent surgical correction of pectus excavatum (PE) between September 2005 and March 2015. Of these patients, 61 patients (33 male and 28 female) were selected to undergo the MOVARPE procedure [11]. The decision to perform the MIRPE or MOVARPE or the Ravitch technique is illustrated in an algorithm (Fig. 1). Patient age ranged from 14 to 45 years (mean 23.4 years). The general indication for surgical repair was based on symptomatic (dyspnea, strain fatigue, or shortness of breath) and/or psychoesthetic disorders. The decision for MOVARPE was based on criteria including age, body shape and height, maturation and development of skeleton and musculature, and extent of deformity or asymmetry. Two patients had undergone a prior intervention with the MIRPE technique; two other patients had previously had a different invasive thoracoplasty during childhood; two suffered from scoliosis; and two patients showed Marfan syndrome. Of the patients, 31 had a symmetric but steep anterior wall inclination and the other 30 showed a considerable asymmetric deformity (Table 1).

**Table 1:** Patient demographics and preoperative characteristics

Characteristic	MOVARPE (*n* = 61)
Period	09/2005–03/2015
Gender, *n* (%)	
Male	33 (54%)
Female	28 (46%)
Age, years (mean)	(14–45) Ø 23.4
Shape, *n* (%)	
Symmetric flat, moderate PE	0
Symmetric deep, severe PE	31 (50.8 %)
Asymmetric	30 (49.2 %)
Preoperative symptoms, *n* (%)	
No symptoms: aesthetic and/or psychoesthetic	29 (47.5%)
Symptoms: fatigue, dyspnea, shortness of breath	32 (52.5%)
Preoperative operation, *n* (%)	
MIRPE	2 (3.3%)
Open heart surgery or thoracoplasty	2 (3.3%)
Connective tissue disorders, *n* (%)	
Marfan syndrome	2 (3.3%)
Scoliosis	2 (3.3%)

*MIRPE* minimally invasive repair of pectus excavatum,* MOVARPE* minor open videoendoscopically assisted repair of pectus excavatum, *PE *pectus excavatum

**Fig. 2:**
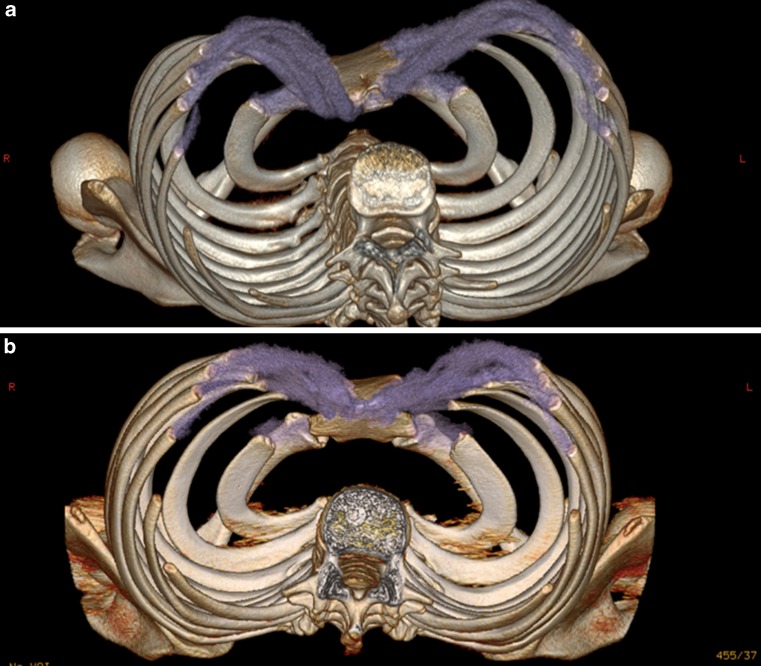
**a** Preoperative CT imaging of a 15-year-old female with an asymmetrical funnel chest. **b** Preoperative CT imaging of a 16-year-old boy with an asymmetric deep funnel chest

Preoperative evaluation included physical examination by ergometry, external measurement of sagittal and transversal chest diameters using a thorax caliper [12], and preoperative three-dimensional volume-rendering mode computed tomography to depict the individual characteristics of any complex deformity (Fig. 2; [13]). Our results were assessed as subjective patient satisfaction according to Davis and Weinstein by two unbiased surgeons who were not involved in patient treatment [14]. Clinical appearance was evaluated as “excellent,” “good,” and “failed” according to the score used by Goretsky et al. [8].

## Surgical technique

**Fig. 3:**
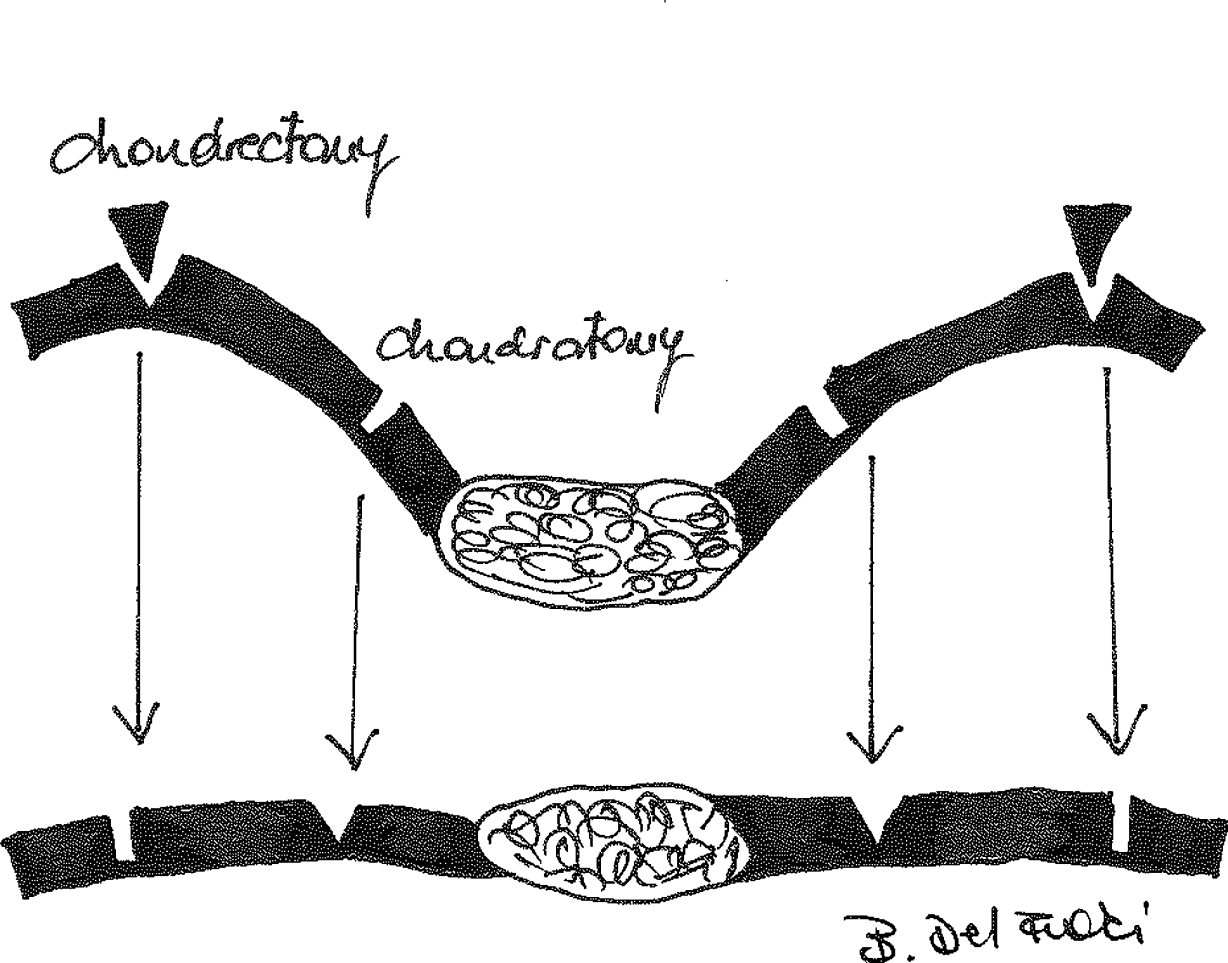
Schematic depiction of a wedge resection in the case of a convex rib and a simple incision in a concave rib

**Fig. 4:**
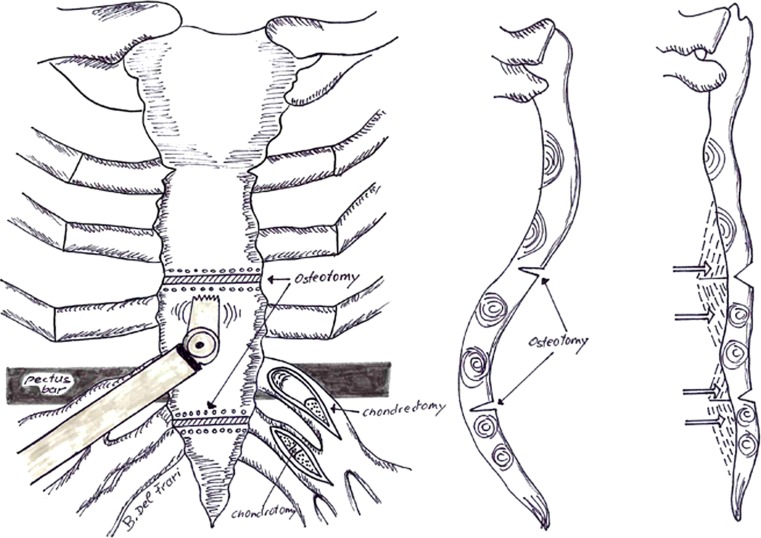
Schematic depiction of the sternum osteotomies with an angled saw, chondrotomies, and partial chondrectomies

Initially, the operation proceeds analogous to the MIRPE procedure [1, 2], with preoperative intravenous antibiotic given as a single dose. Additional incisions are set in the submammary crease in females or in the midline in males. Location and number of distorted ribs are individually selected, depending on the shape and severity of the deformity. Using the split muscle technique, the rib cartilages are incised or partially resected in order to relax the chondrocostal arches [15]. In convex rib arches, a wedge resection is performed; whereas in concave ribs, a simple incision is sufficient to unbend the deformed cartilage curvature (Fig. 3). Depending on the extent of the deformity, more or less partial chondrectomies or chondrotomies are performed. A piezoelectric angled saw is used along a subcutaneous tunnel to perform a transverse sternotomy as a wedge resection (Fig. 4; [10]). While the posterior compacta of the sternum is left uncut in symmetric cases, it has to be completely transected in asymmetric cases in order to correct its malrotation. Such incisions then alleviate elevation of the deformed central thoracic wall unit (Fig. 4). With the aid of a bone hook, the sternum and the anterior thoracic wall—now relaxed thanks to the multiple incisions—are elevated, thus causing intentional green-stick fracture of the posterior sternum compacta. The operation now proceeds as a conventional MIRPE technique with video-assisted thoracoscopy, usually implanting a single pectus bar [1–3]. The lateral wings of the pectus bar are fixed with circumcostal double armed 0‑polydioxanone (PDS) sutures using a Deschamps needle in order to avoid bar displacement [16]. The wings are covered with serratus anterior muscle and wound closure is performed under positive end-expiratory pressure (PEEP) ventilation in order to evacuate surplus air between the pleural layers and eliminate the need for chest tubes. Light activities are allowed 3 weeks and unrestricted sports activities 3 months after surgery.

## Results

**Fig. 5:**
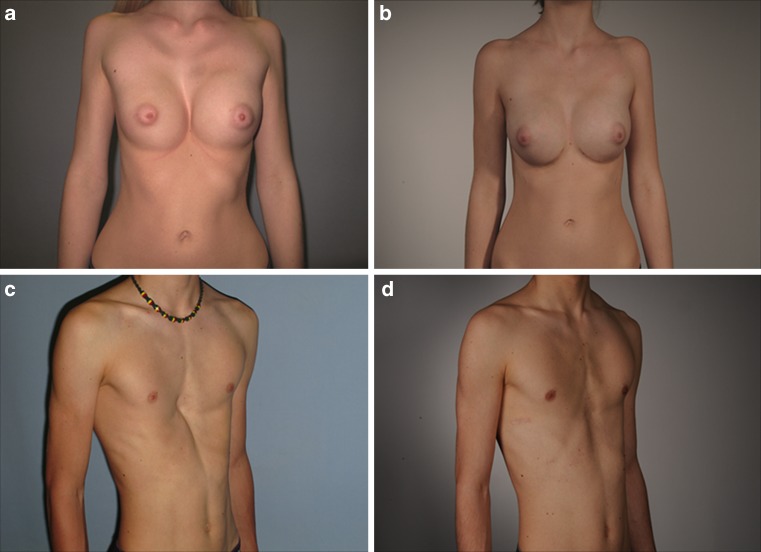
**a** Preoperative frontal view of the 15-year-old female with an asymmetrical funnel chest and distortion of the breast tissue shown in Fig. 2. **b** Postoperative frontal view of the same patient 2 years and 8 months after thoracic wall correction performed with the MOVARPE technique including sternum osteotomy and multiple chondrotomies from the third to the sixth rib. The pectus bar is still in situ. **c** Preoperative right oblique view of the 16-year-old male with an asymmetric deep funnel chest shown in Fig. 2. **d** Postoperative right oblique view of the same patient after PE correction with MOVARPE technique including sternum osteotomy and multiple chondrotomies from the fifth to the seventh rib 1 year after the pectus bar was removed.* PE* pectus excavatum, *MOVARPE* minor open videoendoscopically assisted repair of pectus excavatum

In 61 patients (33 male and 28 female), MOVARPE was successfully performed with a single pectus bar (Fig. 5) implanted in 88.5% and with two bars in 11.5%. Of the patients, 96.7% needed a sternum osteotomy. Bar fixation was performed with circumcostal sutures in 90.2% and with a stabilizer in 9.8%. In 82.3% of the patients in this series the bars were removed after a mean implantation period of 19.3 months (12–35 months). Mean follow-up after bar removal was 13.3 months. According to the score conceived by Goretsky et al. [8] addressing long-term outcome, 76.5% of the results were excellent, 23.5% were good, and there was no failed end result (Table 2). No major intra- or postoperative complications occurred. Minor complications included pleural effusion in four cases, with spontaneous resorption within 3 days. Early pectus bar revision was necessary in two cases to correct tilting of the pectus bar. Mild recurrence of deformity (defined as a minor sunken sternum relapse of 1 to 1.5 cm) after pectus bar removal occurred in only three cases. One case with moderate pneumothorax and respiration impairment required a chest tube (Table 3).

**Table 2:** Long-term results of the 51 patients after bar removal

End result	Patients with bar removed
Excellent^a^	39 (76.5%)
Good^b^	12 (23.5%)
Failed^c^	0 (0.0%)

^a^Deformity is not visible

^b^Deformity is not visible from the front

^c^No improvement of deformity, clearly visible from the front

**Table 3:** Early and late postoperative complications

Characteristic	MOVARPE *n* = 61 (%)
Early	
Pleural effusion	4 (6.5%)
Required chest tube	1 (1.6%)
Pneumothorax	1 (1.6%)
Superficial wound infection	1 (1.6%)
Late	
Mild recurrence after bar removal (*n* – 51)	3 (4.9%)
Bar displacement	2 (3.3%)
Wound infection	1 (1.6%)
Over correction or carinatum deformity	1 (1.6%)

*MOVARPE* minor open videoendoscopically assisted repair of pectus excavatum

## Discussion

Despite the success of the MIRPE procedure in children abundantly reported in the literature [1, 2, 17], extending this procedure to adolescents, adults, athletic persons, and asymmetric cases remains the subject of discussion. In contrast to the setting in children, remodeling a rigid, stiff chest wall using a single or two pectus bars is exceptionally challenging. Technical intricacy during the surgical procedure, increased rates of ensuing complications [18–20], higher rates of pain proportional to the pressure applied to the thoracic skeleton [21], and a higher risk of an undesired outcome [20] and recurrence [2, 17, 22] are likely to be encountered due to lack of tissue pliability in cases with matured skeletal structures, severe deformities, and, particularly, in asymmetric cases with sternum malrotation.

The decision for the appropriate surgical technique—either MIRPE or MOVARPE—depends on age, as well as the shape of the funnel deformity itself and the patient’s physique. For this reason, an algorithm for correcting pectus excavatum deformities is applied in our department [10].

In a meta-analysis, Nasr et al. compared the two most often applied techniques, i.e., the Nuss and Ravitch procedures, and found no significant differences with respect to overall complications and length of hospital stay, although the rates of reoperation and hemo- and pneumothorax were higher in the Nuss procedure [23]. Therefore, it appears to be constructive to combine the advantages of both techniques in the MOVARPE approach, consisting of a conventional osteochondrotomies hybridized with the videoendoscopically assisted implantation of a pectus bar [9]. In contrast to conventional open surgery, this approach is accomplished with rather small surgical incisions and minor surgical trauma, but provides stable support of the remodeled thoracic wall until the skeletal structures have completely healed. Osteochondrotomies or partial chondrotomies reduce immediate- as well as long-term postoperative pain caused by diminished lever forces of the pectus bar against the posterior sternum periosteum [24]. Elevation and remodeling of the concave anterior thoracic wall to a natural convexity by twisting the intrathoracally placed pectus bar is facilitated by the relaxing osteochondromoties, in contrast to elevation achieved by pressure forces alone as in MIRPE. In 2009, Al-Assiri et al., studying a collective of 15 children, already stated that sternocostal “relaxing” incisions in the cartilaginous portion of the ribs, in addition to the standard MIRPE technique, appear to facilitate retrosternal dissection and reduce postoperative chest wall tension [25]. The time allotted for healing of the sternotomy and chondroplasties is only 2 to 3 months, and the necessary support afforded by the pectus bar is reduced to only 2 years versus a period of up to 4 years implantation time [17] for the MIRPE chest wall elevation technique that is based merely on bone and cartilage distension. Osteotomized sternum bone and relaxing chondrotomies usually heal with rapid stable callus and scar formation, thus permitting pectus bar removal much earlier than in the cases solely employing distension [17]. Lever support by a pectus bar alone has to override the memory properties of the elasticity of a various number of deformed cartilage arches, as well as the bent sternum bone, over a long period [24, 26]. In the rare cases using a second pectus bar, particularly in adolescents or adults with athletic body shape or very tall patients, the decision for the second bar is made intraoperatively, based on the lever force remaining after the osteochondrotomies are performed.

In addition to our results reported in a prior publication, the results of this new 10-year study without any failed end results also confirm MOVARPE as a rational approach in select patients [10]. Minor drawbacks of MOVARPE versus MIRPE, consisting of a prolonged operating time of up to 1 hour and additional scars, did not bother any patient in this series. The greater surgical effort and additional minor scars nevertheless appear to be justified with regard to the intra- and postoperative procedural advantages and final long-term outcome.

## Conclusion

Our experience shows that both MIRPE and MOVARPE are appropriate techniques, but the decision on which technique to apply is based on different selection criteria. In general, MIRPE is still used as a standard procedure for uncomplicated cases and remains an ideal therapeutic option in childhood and adolescence with symmetric pectus excavatum deformities. MOVARPE, on the other hand, is a complementary method with high efficacy for the correction of already matured rigid skeletal structures at or beyond puberty, and for complex pectus excavatum deformities.
